# The effectiveness of German disease management programs (DMPs) in patients with type 2 diabetes mellitus and coronary heart disease: results from an observational longitudinal study

**DOI:** 10.1186/s13098-015-0065-9

**Published:** 2015-09-17

**Authors:** Michael Laxy, Renée Stark, Christa Meisinger, Inge Kirchberger, Margit Heier, Wolfgang von Scheidt, Rolf Holle

**Affiliations:** Helmholtz Zentrum München, Institute of Health Economics and Health Care Management, Ingolstädter Landstraße 1, 85764 Neuherberg, Germany; German Center for Diabetes Research, Ingolstädter Landstraße 1, 85764 Neuherberg, Germany; Helmholtz Zentrum München, Institute of Epidemiology II, Ingolstädter Landstraße 1, 85764 Neuherberg, Germany; MONICA/KORA Myocardial Infarction Registry, Central Hospital of Augsburg, Stenglinstr. 2, 86156 Augsburg, Germany; Department of Internal Medicine I-Cardiology, Central Hospital of Augsburg, Stenglinstr. 2, 86156 Augsburg, Germany

**Keywords:** Diabetes, Coronary heart disease, Disease management programs, Guideline care, (Quality-adjusted) survival, Self-management

## Abstract

**Background:**

Although the population-based German disease management programs (DMPs) for diabetes mellitus (DM) and coronary heart disease (CHD) are among the biggest worldwide, evidence on the effectiveness of these programs is still inconclusive or missing, particularly for high risk patients with comorbidities. The objective of this study was therefore to analyze the impact of DMPs on process and outcome parameters in patients with both, type 2 DM and CHD.

**Methods:**

Analyses are based on two postal surveys of patients from the KORA myocardial infarction registry (southern Germany) with type 2 DM and on two postal validation studies with patients’ general physicians (2006, n = 312 and 2011, n = 212). The association between DMP enrollment (being enrolled in either DMP-DM or DMP-CHD) and guideline care (defined by several process indicators) at baseline (2006) and its development until follow-up (2011) was analyzed using logistic regression models accounting for the repeated measurements structure. The impact of DMP enrollment/guideline care on cumulated (quality-adjusted) life years ((QA)LYs) over a 4-year time horizon (2006–2010) was assessed using multiple linear regression methods. Logistic regression models were applied to analyze the association between DMP status and patient self-management at follow-up.

**Results:**

Being enrolled in a DMP was associated with better guideline care at baseline [OR = 2.3 (95 % CI 1.27–4.03)], but not at follow-up [OR = 0.80 (95 % CI 0.40–1.58); *p* value for time-interaction <0.01]. DMP enrollment was not significantly [+0.15 LYs (95 % CI –0.07, 0.37); +0.06 QALYs (95 % CI –0.15, 0.26)], but treatment according to guideline care significantly [+0.40 LYs (95 % CI 0.21–0.60); +0.28 QALYs (95 % CI 0.10–0.45)] associated with higher (quality-adjusted) survival over the 4-year follow-up period. DMP enrollees further reported a somewhat better self-management than patients not being enrolled into a DMP.

**Conclusions:**

The results of this study concerning the effectiveness of DMPs in patients with DM and CHD are mixed, but are weakly in favor of DMPs. However, we found a clear positive impact of guideline care on quality adjusted survival in this patient group. The development of the association between DMP enrollment and guideline care over the follow-up time indicates some external effects, which should be the subject of further investigations.

**Electronic supplementary material:**

The online version of this article (doi:10.1186/s13098-015-0065-9) contains supplementary material, which is available to authorized users.

## Background

Metabolic and cardiovascular diseases (CVD) are interrelated public health problems causing substantial economic pressure on health care systems [1, 2]. As the prevalence of diabetes mellitus (DM) is currently rising in Germany and in most other countries and as diabetes is a major risk factor for CVD, the burden of these diseases is expected to increase further [3–5]. Specifically, high risk individuals with co-existing cardiovascular and metabolic diseases need intensive treatment to prevent macro-vascular complications. Owing to their similar pathogenesis [6], treatment guidelines in both diabetes and coronary heart disease (CHD) focus on the management of CVD risk factors through modification of lifestyle habits and medication therapy as these strategies have been shown to improve patient outcomes [7–13]. In order to reinforce guideline care and to improve quality of care for chronically ill patients, in the years 2002/2003, disease management programs (DMPs) for DM and CHD were rolled out nationwide in Germany within the statutory health insurance (SHI) system, a system of non-profit health insurance companies insuring ~88 % of the population [14]. In comparison to disease management programs in other countries which often focus on high risk patients and are heterogeneous in their structure, German DMPs are population-based approaches and characterized by a high degree of homogeneity. In Germany, the “Federal Joint Committee” (Gemeinsamer Bundesausschuss) which is responsible for decision-making and quality assurance in the inpatient and outpatient health care sector passes directives and content requirements for structured DMPs [15]. All DMPs must obtain approval from the German “Federal Insurance Agency” (Bundesversicherungsamt), which controls the quality standards passed by the Federal Joint Committee [14, 16]. Within these programs, general practitioners (GPs) act as gatekeepers responsible for (voluntary) enrollment of eligible patients and coordination of care. The key contents of the DMP-DM and DMP-CHD are enforcement of medication therapy, enhanced patient activation and patient self-management education (e.g., diet, physical activity, smoking cessation), continuity of care according to current guidelines and the use of information technology systems for routine documentation/benchmarking [16]. Details including the roles of GPs, patients and health insurances as well as reimbursement schemes have been described elsewhere [17, 18]. Although around 4.0 million patients with type 2 DM and 1.8 million patients with CHD are enrolled in the respective German DMPs, a scientifically sound evaluation of the programs has appeared to be difficult [16]. Important clinical information is only routinely collected for DMP participants, but not for non-DMP patients, and running randomized trials is hardly possible, as by law patients have the right to be enrolled into the appropriate programs [19, 20]. Therefore, the evidence on the effectiveness of DMPs mainly relies on non-randomized group comparisons based on a limited subset of information from routine claims [17, 18, 21, 22] or population-based survey data [23–28]. These studies show mixed results on survival and better quality of care for patients enrolled in the respective DMPs. However, due to the population-based focus of German DMPs, no study has examined the effectiveness of DMPs in terms of process and outcome quality in multi-morbid high risk patients. In addition, although patient education is one of the key DMP contents [16, 29, 30], very little is known about the impact of these education programs on patient self-management.

This study aims to evaluate the effectiveness of German DMPs in patients with coexistence of diabetes and CHD using longitudinal follow-up data from a registry-based population (myocardial infraction registry). In detail, we analyzed the impact of DMP enrollment (either DMP-DM or DMP-CHD) on guideline care, the impact of DMP enrollment/guideline care on 4-year (quality-adjusted) survival, and on patient self-management.

## Methods

### Data source

**Fig. 1:**
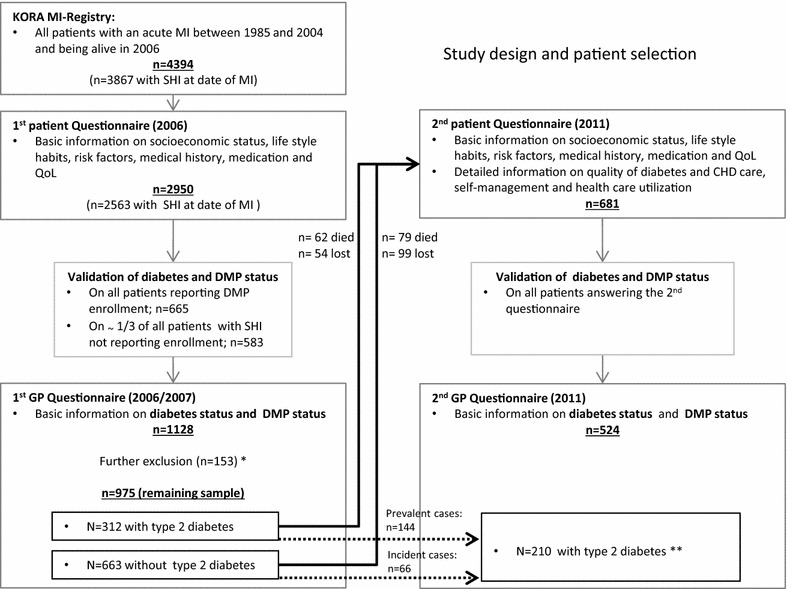
Study design and patient selection. *DMP status not reported by physician (n = 29), patient not cared for by physician (n = 31), patient is privately insured (n = 79), patient not eligible for DMP (n = 8), patient questionnaire empty (n = 6). **3 inconsistent cases (diabetes type 2 indication in 2006, but not in 2011) were excluded

Data for this study originated from the core documentation of the KORA (Cooperative Health Research in the Region of Augsburg) myocardial infarction (MI) registry and two postal questionnaire waves (2006 and 2011) in MI registry patients and their respective GPs. The KORA MI registry collects information on all cases of coronary deaths and non-fatal MIs of inhabitants aged 25–74 years in the city of Augsburg and two surrounding counties [31]. Of the 2950 participants who were alive in 2006 (n = 4394, response ~67 %) and answered an initial postal survey (patient survey 1) assessing basic information on socioeconomic status, lifestyle habits, risk factors, medication, and quality of life (QoL), 2563 were insured in the SHI system at the date of MI. As DMP names vary considerably between statutory health insurance companies and may not reveal the disease management aspect, the information about the DMP status was assessed from the patients’ GPs. For this, all GPs of statutorily insured participants who reported DMP-CHD enrollment (n = 665) and a subsample of the GPs of statutorily insured patients who denied DMP-CHD enrollment (n = 583) were asked to provide information on the DMP status of their patients (validation survey 1). The GPs of 1128 patients answered this questionnaire. For several reasons, 153 patients were excluded from further study (details in Fig. 1). Of the remaining 975 patients, 312 were identified as having type 2 diabetes. Information on the diabetes status and the type of diabetes was extracted from the GP questionnaires. If the physicians’ information on diabetes status was missing, the information provided by the patient was used (sensitivity 80 %; specificity 99 %). Up to the end of 2010, 141 patients died (62 of those with type 2 diabetes) and 681 of the patients still alive answered a second postal questionnaire in 2011 (patient survey 2), assessing the same variables as in 2006, plus information on patient self-management behavior. As in 2006, GPs were contacted again in 2011, and information on diabetes and DMP status was retrieved for 524 patients through a postal questionnaire (validation survey 2). Of those 524 patients, 210 were identified as having type 2 diabetes; 144 already had a diagnosis in 2006 and 66 were incident cases between 2006 and 2011. Only patients with physician-validated information on DMP status (n = 312 in 2006 and n = 210 in 2011) were included in the analysis, as previous analyses have shown that the validity of patient self-reports on their DMP status is low [32]. The detailed study design and patient selection are illustrated in Fig. 1. Data collection and analyses related to the registry have been approved by the Bavarian Ethics Committee.

### Measures

#### DMP enrollment

Information about the patients’ DMP enrollment status was extracted from the GP questionnaire in 2006 and 2011. All patients in the analyzed sample were eligible for enrollment in both DMP-CHD and DMP-DM. Therefore, a patient was classified as being a DMP enrollee if enrollment in one or in both of these programs was reported.

#### Guideline care

Treatment of patients in the DMP-CHD and DMP-DM should be based on national treatment guidelines [16], which include among other criteria, counseling for the topics physical activity, diet, and smoking cessation and treatment with statins, platelet aggregation inhibitors (PAI), and antihypertensive drugs [either renin–angiotensin inhibitors (RAI) or beta blocker] [33, 34]. As in a previous study, we defined guideline care as being present if: (a) a patient had received medical advice over the last 12 months on at least two out of the three lifestyle topics (smoking, diet, exercise) and (b) reported the intake of a PAI and a statin and either RAI or a beta blocker over the last 7 days [27].

#### Quality of life, survival, and quality-adjusted survival

Information on the vitality status of patients was obtained by address search and by contacting the regional registration authorities. Survival times started at the fill-in date of the patient’s questionnaire in 2006 and ended at date of censoring or death. Quality of life was assessed in both patient questionnaires (2006 and 2011) using the EQ-5D instrument and utility values were calculated using a scoring algorithm derived from the general German population [35]. Combining survival times and QoL, accumulated (quality-adjusted) life years ((QA)LYs), a measure that accounts for both the length and the quality of life over a certain observation period, were assessed individually for each person according to the methodology described by Laxy et al. [36].

#### Self-management

Patient self-management was defined by means of previous established criteria [26, 37–40]. The binary indicators, comprised of regular moderate physical activity (≥5 h per week) [40] and regular (at least once a week) self-monitoring of blood glucose (SMBG), blood pressure (SMBP), and body weight (SMBW), were extracted from the patients’ self-reports [26, 37, 38]. Medication adherence was assessed according to the German version of the Morisky Medication Adherence Scale (MMAS-4 score <1) [39]. As an indicator of patient knowledge, it was asked if patients knew about the term “HbA1c” as a performance measure for long-term glucose control. Furthermore, it was assessed whether patients participated in an education class for diabetes or blood pressure control.

### Statistical analysis

Descriptive statistics of the samples’ characteristics in 2006 (n = 312) and 2011 (n = 210) and p-values (Chi square tests for categorical variables and t-tests for continuous variables) for the differences between DMP enrollees and non-enrollees and between patients receiving guideline vs. those not receiving guideline care are reported.

In order to test whether DMP enrollment is a predictor for receiving guideline care and to test how this association developed over time, we used a generalized estimating equation (GEE) model with a logit-link, which accounts for the partly repeated measurement structure of the sample and included an interaction term between DMP status (yes vs. no) and time (2006 vs. 2011). Linear regression and Cox proportional hazard regression was applied to test the impact of the DMP status/guideline care on QoL changes and mortality over the 4-year follow-up period (2006–2010). Differences in accumulated (quality-adjusted) life years over the follow-up period for DMP status/guideline care were tested through linear regression in order to obtain an absolute measure for the achieved health gains. Finally, we applied logistic regression to assess the cross-sectional association between DMP/guideline care and the patients’ self-management in 2011. A more sophisticated analysis of the association with self-management was not possible, as self-management was only assessed in the 2011 survey.

As part of our additional exploratory analyses, we further examined separately the impact of DMP-DM and DMP-CHD to capture potential differences in the effectiveness of these programs.

All models were adjusted for the covariates age (continuous), sex, educational status (primary education: ≤9 years of school; secondary and tertiary education: >9 years of school), smoking status (never smoker, ex-smoker, current smoker), weight status (normal weight: Body Mass Index (BMI) < 25; overweight: 25 ≤ BMI < 30; obese: BMI ≥ 30), number of re-infarctions in 2006 (continuous), treatment status (no medication, oral antidiabetic medication only, insulin therapy with or without oral antidiabetic medication) and diabetes duration (<5 years, 5≤ years <10 years, ≥10 years), which were extracted from self-reports of the surveys in 2006 and 2011 and from the core database of the MI registry. Linear regression models analyzing the difference in accumulated QALYs were also adjusted for QoL at baseline (2006). For each association, a separate regression model was built up including only the predictor of interest and the specified set of covariates.

## Results

### Characteristics of the study population

The characteristics of the 2006 and 2011 sample are shown in Table 1. The mean age of the sample was 68.2 years in 2006 and 72.1 years in 2011. Mean diabetes duration (mean duration since the first registry-registered MI) averaged ~9 (~9) years in 2006 and ~10 (~14) years in 2011. As patients originate from a MI registry, almost 80 % of people were male. In 2011, 77 % (2006, 76 %) were enrolled in one of the two DMPs, and 61 % (2006, 62 %) were treated according to guideline care. DMP enrollees and non-enrollees were quite comparable but patients receiving guideline care were on average significantly younger and reported higher QoL values than those not receiving guideline care.

**Table 1:** Baseline characteristics of patients in 2006 and 2011 according to their enrolment into DMPs and according to guideline care

	2006 (baseline)								2011 (follow-up)							
	n = 312		DMP (CHD or DM)			Guideline care^a^			n = 210		DMP (CHD or DM)			Guideline care^a^		
			Yes (n = 237)	No (n = 75)		Yes (n = 186)	No (n = 114)				Yes (n = 161)	No (n = 49)		Yes (n = 120)	No (n = 76)	
	Mean	Std	Mean	Mean	p-value	Mean	Mean	p-value	Mean	Std	Mean	Mean	p-value	Mean	Mean	p-value
Age	68.2	8.6	67.7	69.9	0.052	67.1	69.3	0.033	72.1	8.3	71.6	73.7	0.116	70.4	74.3	0.001
Diabetes duration (years)	8.5	7.5	8.5	8.7	0.838	8.7	8.2	0.661	9.9	7.0	10.7	7.2	0.006	10.2	8.5	0.144
Time since first MI	9.3	5.3	8.7	11.0	0.001	9.0	9.9	0.123	14.1	5.2	14.1	14.1	0.956	13.6	15.0	0.069
Number of reinfarctions	0.2	0.5	0.2	0.2	0.233	0.2	0.1	0.073	0.1	0.4	0.1	0.1	0.163	0.1	0.1	0.347
Quality of life (EQ-5D)	0.81	0.23	0.82	0.80	0.474	0.84	0.77	0.007	0.81	0.23	0.81	0.78	0.466	0.83	0.78	0.099

	n	%	%	%	p-value	%	%	p-value	n	%	%	%	p-value	%	%	p-value
Sex																
Women	64	20.5	49	15	0.900	37	23	0.953	44	21.0	37	7	0.190	20	18	0.226
Men	248	79.5	188	60		149	91		166	79.1	124	42		100	58	
Education																
Low (<9 years of school)	221	77.8	177	44	0.025	133	80	0.596	152	77.6	116	36	0.895	91	51	0.666
High (≥9 years of school)	63	22.2	42	21		41	21		44	22.5	34	10		26	17	
Smoking																
Never	92	29.9	66	26	0.459	55	31	0.851	70	33.8	52	18	0.846	39	25	0.552
Ex-smoker	179	58.1	138	41		105	69		117	56.5	90	27		67	43	
Current smoker	37	12.0	30	7		23	14		20	9.7	16	4		14	5	
Weight status																
Normal (20 ≤ BMI < 25)	37	12.2	26	11	0.484	20	16	0.461	30	14.9	18	12	0.092	11	16	0.041
Overweight (25 ≤ BMI < 30)	146	48.0	115	31		91	47		91	45.1	71	20		59	28	
Obese (BMI ≥ 30)	121	39.8	90	31		72	46		81	40.1	64	17		47	30	
Diabetes treatment																
No medication	59	19.7	41	18	0.418	35	21	0.995	56	27.3	41	15	0.617	33	21	0.955
Oral medication only	140	46.7	108	32		86	51		96	46.8	74	22		56	35	
Insulin therapy	101	33.7	79	22		59	36		53	25.9	43	10		30	17	
Guideline care^a^																
No	114	38.0	76	38	0.001	–	–	–	76	38.8	59	17	0.876	–	–	–
Yes	186	62.0	154	32		–	–		120	61.2	92	28		–	–	

*DMP* disease management program, *CHD* coronary heart disease, *DM* diabetes mellitus

^a^Counseling on two out of the three lifestyle topics (smoking, diet, exercise) and intake of a platelet aggregation inhibitor and a statin and either renin–angiotensin inhibitors or a beta blocker

### Association between DMP status and guideline care

Table 2 depicts the association between DMP status and guideline care and how this relationship changed from 2006 to 2011. In 2006, patients enrolled in one of the two DMPs were 2.3 times (95 % CI 1.27–4.03) more likely to receive guideline care than patients not enrolled in a DMP. However, although the portion of DMP enrollees receiving guideline care remained relatively stable until 2011, the portion of non-DMP enrollees increased substantially, resulting in a non-significant association between DMP enrollment and guideline care (OR = 0.80, 95 % CI 0.40–1.58). The interaction term for this effect was highly significant (p = 0.009). A similar pattern was observed for enrollment in DMP-CHD or DMP-DM only.

**Table 2:** Adjusted odds ratios (OR) on the association between DMP status and guideline care in 2006 and 2011 with an interaction term DMP status *x* time

Treatment status	General estimating equation model with logit link						DMP status *x* time p-value
	Guideline care in 2006^a^			Guideline care in 2011^a^			
	%	OR	(95 % CI)	%	OR	(95 % CI)	
DMP (CHD or DM)							
No	45.7	Ref.		62.2	Ref.		
Yes	67.0	2.27	(1.27–4.03)	60.9	0.80	(0.40–1.58)	0.009
DMP-CHD (only)							
No	54.7	Ref.		62.1	Ref.		
Yes	69.3	1.91	(1.17–3.14)	60.8	0.92	(0.50–1.70)	0.035
DMP-DM (only)							
No	55.4	Ref.		59.0	Ref.		
Yes	66.0	1.48	(0.88–2.46)	62.7	0.96	(0.52–1.76)	0.231

Models adjusted for age, sex, education, smoking status, weight status, treatment status, number of reinfarctions, and diabetes duration

*DMP* disease management program, *CHD* coronary heart disease, *DM* diabetes mellitus

^a^Counseling on two out of the three lifestyle topics (smoking, diet, exercise) and intake of a platelet aggregation inhibitor and a statin and either renin–angiotensin inhibitors or a beta blocker

### Association between DMP status/guideline care and (quality-adjusted) survival

Table 3 shows the relationship between DMP status/guideline care and QoL changes and mortality respectively. Effect estimates from the linear regression model show that neither DMP enrollment nor guideline care was significantly associated with QoL change over the follow-up period. The results from the Cox proportional hazard regression show that enrollment in a DMP was not significantly associated with mortality (HR = 0.80; 95 % CI 0.45–1.41). Separate analyses for DMP-CHD (HR = 0.74; 95 % CI 0.44–1.25) and DMP-DM (HR = 0.91; 95 % CI 0.54–1.54) also showed non-significant associations.

**Table 3:** Adjusted hazard rations (HR) and adjusted mean differences on the association between DMP status/guideline care and mortality/QoL change

Treatment status in 2006	Cox regression model		Linear regression model	
	Mortality		Change in EQ-5D per year	
	HR	(95 % CI)	Beta	(95 % CI)
DMP (CHD or DM)				
No	Ref.		Ref.	
Yes	0.80	(0.45 to 1.41)	−0.009	(−0.029 to 0.011)
Guideline care^a^				
No	Ref.		Ref.	
Yes	0.27	(0.15 to 0.47)	−0.003	(−0.021 to 0.014)
DMP-CHD (only)				
No	Ref.		Ref.	
Yes	0.74	(0.44 to 1.25)	0.003	(−0.013 to 0.019)
DMP-DM (only)				
No	Ref.		Ref.	
Yes	0.91	(0.54 to 1.54)	0.002	(−0.014 to 0.019)

Models adjusted for age, sex, education, smoking status, weight status, treatment status, number of reinfarctions, and diabetes duration

*DMP* disease management program, *CHD* coronary heart disease, *DM* diabetes mellitus, *QoL* quality of life

^a^Counseling on two out of the three lifestyle topics (smoking, diet, exercise) and intake of a platelet aggregation inhibitor and a statin and either renin–angiotensin inhibitors or a beta blocker

Treatment according to guideline care was also not associated with QoL change, but was a very strong predictor of mortality (HR = 0.27; 95 % CI 0.15–0.47). More detailed analyses on the single dimensions of guideline care are illustrated in Additional file 1: Appendix 1. All dimensions were associated with a significant or non-significant mortality risk reduction (advice diet, HR = 0.57, p = 0.12; advice exercise, HR = 0.65, p = 0.15; beta-blockers, HR = 0.27, p < 0.001; statins, HR = 0.40, p < 0.01; PAI, HR = 0.47, p = 0.05; RAI = 0.69, p = 0.10).

Table 4 shows the relationship between DMP status/guideline care and (quality-adjusted) survival. Enrollment in a DMP was not significantly associated with accumulated LYs (+0.15, 95 % CI −0.07 to 0.37) and QALYs (+0.06; 95 % CI −0.15 to 0.26). Additional analyses showed that DMP-CHD was (borderline) significantly associated with accumulated LYs (β = 0.21, 95 % CI 0.02–0.40) and QALYs (β = 0.16, 95 % CI 0.00–0.33). The discrepancy between the non-significant HR and the (borderline) significant absolute effect estimates (LYs, QALYs) for DMP-CHD status results mainly from a shorter survival time of people who die, which is not accounted for by the non-parametric Cox model.

**Table 4:** Adjusted mean differences on the association between DMP status/guideline care and accumulated LYs/QALYs over the 4-year follow-up period

Treatment status in 2006	Linear regression model			Linear regression model		
	Difference in accumulated LYs^a^			Difference in accumulated QALYs^b^		
	Adj. mean	Beta	(95 % CI)	Adj. mean	Beta	(95 % CI)
DMP (CHD or DM)						
No	3.64	Ref.		2.95	Ref.	
Yes	3.78	0.15	(−0.07 to 0.37)	3.01	0.06	(−0.15 to 0.26)
Guideline care^c^						
No	3.51	Ref.		2.83	Ref.	
Yes	3.91	0.40	(0.21 to 0.60)	3.11	0.28	(0.10 to 0.45)
DMP-CHD (only)						
No	3.65	Ref.		2.92	Ref.	
Yes	3.86	0.21	(0.02 to 0.40)	3.08	0.16	(0.00 to 0.33)
DMP-DM (only)						
No	3.70	Ref.		2.98	Ref.	
Yes	3.78	0.08	(−0.11 to 0.28)	3.00	0.02	(−0.16 to 0.20)

*DMP* disease management program, *CHD* coronary heart disease, *DM* diabetes mellitus, *LYs* (unadjusted) life years, *QALYs* quality adjusted life years

^a^Models adjusted for age, sex, education, smoking status, weight status, treatment status, number of reinfarctions, and diabetes duration

^b^Models adjusted for (a) and QoL at baseline

^c^Counseling on two out of the three lifestyle topics (smoking, diet, exercise) and intake of a platelet aggregation inhibitor and a statin and either renin–angiotensin inhibitors or a beta blocker

Guideline care was strongly associated with longer survival (+0.40 LYs, 95 % CI 0.21–0.60) and quality-adjusted survival (+0.28 QALYs, 95 % CI 0.10–0.45) over a time frame of ~4 years. Detailed analyses showed, that particularly patients taking beta-blockers (+0.29 QALYs, 95 % CI 0.06–0.52), statins (+0.25 QALYs, 95 % CI 0.04–0.46), and PAI (+0.25 QALYs, 95 % CI 0.04–0.46) had a substantially higher quality-adjusted survival over the follow-up period.

### Association between DMP status/guideline care and self-management

**Fig. 2:**
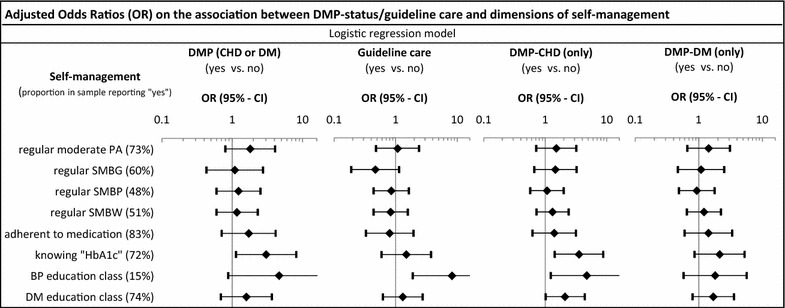
Adjusted odds ratios (OR) on the association between DMP-status/guideline care and dimensions of self-management. *DMP* disease management program, *CHD* coronary heart disease, *DM* diabetes mellitus, *PA* physical activity, *SM* self‐monitoring, *BG* blood glucose, *BP* blood pressure, *BW* body weight, *HbA1c* glycated hemoglobin; *Guideline care* counselling on 2 out of the 3 lifestyle topics (smoking, diet, exercise) and intake of a platelet aggregation inhibitor and a statin and either a renin angiotensin inhibitors or a beta blocker; models adjusted for age, sex, education, smoking status, weight‐status, treatment status, number of re‐infarctions and diabetes duration; exact point estimates and 95 % CI are reported in Additional file 2: Appendix 2

Figure 2 shows the association between DMP/treatment status and important dimensions of self-management. Overall, patients enrolled in a DMP reported better self-management (almost all OR >1); however, only patient knowledge (“knowing the term HbA1c”) (OR: 3.05, 95 % CI 1.13–8.20) was significantly associated with DMP enrollment. Separate analyses for DMP-DM and DMP-CHD showed a similar pattern. Overall, the uncertainty around effect estimates was large.

## Discussion

This is the first study in Germany and one of the first studies internationally evaluating the effect of structured DMPs in individuals with both DM and CHD, a patient group known to have a high risk of fatal and non-fatal cardiovascular events [29, 41]. In addition, to our knowledge it is the only study in Germany on DMPs which uses data after 2007 [26, 29]. Overall, we found mixed results for the association between DMP enrollment and patient outcomes in high risk patients, which are weakly in favor of DMPs. DMP enrollment was positively associated with guideline care at the beginning of the study (2006), but not any longer the end of the study (2011). Although guideline care at baseline (2006) was identified as a strong predictor for (quality-adjusted) survival, we found no evidence for an impact of DMP enrollment on (quality-adjusted) survival over a time period of 4 years. In contrast, DMPs were weakly associated with better patient self-management.

At baseline (2006) the likelihood of receiving guideline care was more than doubled for those patients enrolled in a DMP for CHD and/or DM. This is congruent with previous studies, which clearly have shown that DMPs are associated with improvements in process parameters such as medical examinations, lifestyle counseling, and medication for secondary prevention of CHD [26–28, 42]. Interestingly, although the prevalence of receiving guideline care in the group of DMP enrollees stayed constant or went down slightly, the prevalence of receiving guideline care in the non-DMP group in this study went up substantially from 2006 to 2011. This significant interaction between the effects of DMP and time could be an indicator of a spill-over effect. It is possible that, with the implementation of DMPs and corresponding physician education requirements, physicians started to apply the adopted standards to all treated patients. However, alternative explanations such as altered patterns of enrollment regarding patient characteristics (education, socioeconomic status) or other external effects might be possible, as well.

The results of our study further show that general DMP enrollment in high risk patients was weakly, but non-significantly associated with (quality-adjusted) survival. Previous studies based on claims data from the BARMER (n ~40,000, mortality: RR = 0.48) and the AOK (n ~4000, HR = 0.68) health insurances companies reported significant mortality risk reductions for general diabetes patients enrolled in a DMP-DM [18, 24]. However, our non-significant results are in line with a study by Stark et al. on all participating CHD patients (with and without diabetes) from the same registry-based data source, which showed a moderate but non-significant mortality risk reduction (n = 975, HR = 0.90) for patients being enrolled in a DMP-CHD [27]. In Stark et al.’s study, guideline care was found to be a strong predictor of mortality in CHD patients (HR = 0.40). Our study shows that this association is even stronger in high risk CHD patients with metabolic comorbidity (HR = 0.28), resulting in a 0.28 QALYs longer quality-adjusted survival over a 4-year time frame. The main driver for this strong effect in our study was medication with beta-blockers, statins, and PAI indicating that secondary prevention according to current German national guidelines might prolong life expectancy at a high level of QoL and should therefore have a high priority in the disease management process. Beyond medication and regular check-ups, involvement and education of patients aiming to enhance self-management behavior are core contents of the DMPs [16, 17]. This study shows that at follow-up patients with diabetes and CHD who were enrolled in a DMP reported slightly better self-management; however, owing to a high magnitude of uncertainty, most of these associations were not significant. These findings contribute to the very limited literature on this topic. The only German study that analyzed the association between DMP enrollment and self-management was based on diabetes patients only and reported that DMP enrollees were more likely to have participated in a diabetes education class, however, behavioral aspects such as self-monitoring of feet, blood glucose, or weight were also not significantly improved [26]. As previous research indicated that self-management is a crucial factor for intermediate and long-term health outcomes, future studies evaluating the effectiveness of DMPs should incorporate dimensions of self-management as separate outcome measures to improve the evidence on this important topic [38, 43].

Our exploratory analyses indicated that both programs, DMP-DM and DMP-CHD, were associated with slightly better outcomes in this high risk population. Although some effects were slightly stronger for DMP-CHD, there is not enough evidence to assume that these differences occurred systematically.

The strong association between guideline care and (quality-adjusted) survival and the diminishing association between DMP enrollment and guideline care over the follow-up time are of great (political) relevance when it comes to the discussion about the meaningfulness of German DMPs: given the assumption that positive effects of DMPs are translated through delivery of guideline care, it is likely that the positive impact of DMPs on survival that had been reported in studies on data before 2007 will have been attenuated in recent years. Contrarily, one can argue that in case of a potential treatment spill-over the implementation of DMPs was an effective strategy for improving health care and health outcomes on the population level. Due to its moderate sample size and potentially limited generalizability this study might not provide enough evidence to derive externally valid conclusions on this question, however, the results of this study can be seen as an important guidepost for future research. To answer the question about the overall effectiveness of German DMPs in normal and high-risk patients, larger studies with up to date data are needed. Linking registry-based or population-based survey data, comprising detailed information on behavioral and clinical parameters, with administrative claims data, comprising detailed information about delivered care, seems to be a promising approach in this context [44].

The strength of this study is its registry-based longitudinal design and the analysis of different disease management dimensions. The use of patient self-reports and physician-validated information on DMP status at baseline and follow-up made it possible to comprehensively analyze the association between DMP status, guideline care, mortality, and self-management over the follow-up period. In addition, parallel to classical Cox proportional hazard regression, we applied a method that allows the quantification of quality-adjusted survival based on individual-level longitudinal survey data [36]. This approach has two advantages. First, it not only accounts for the length of life, but also for the health state people live in. This is particularly of interest in older multi-morbid populations where health gains (longer survival) are often achieved through intensive treatment regimens, which in turn might negatively affect QoL. Second, absolute measures for health gains are generally needed for the assessment of the cost-effectiveness of interventions. Taking the means and standard errors of published effects (QALYs) and a certain societal willingness to pay threshold allows one to roughly estimate up to which costs DMPs and guideline care would be considered to be cost-effective.

The major limitation of this study is its non-randomized observational design. Therefore, selection bias and, consequently, effects of reverse causation cannot be ruled out. One big source of selection bias might be the inclusion criteria of German DMPs themselves, in which it is stated that patients need to be willing to actively participate in the DMP [16]. Consequently, it could be assumed that better educated and more active patients with fewer comorbidities were more likely to be enrolled in one of the DMPs. Previous studies have reported such selection effects. Although we could not observe such effects in our data, unobserved selection bias could have occurred resulting in overoptimistic effect estimates [22, 28, 45]. Also, the strong association between guideline care and mortality might be partly attributable to selection processes. It is plausible that severely ill patients with low life expectancy or other severe illnesses such as cancer are treated less “aggressively” with medication for the secondary prevention of CHD. In contrast, we also have some indication for dilution of the observed positive effects: We do know that many of the patients initially not enrolled in a DMP were subsequently enrolled after the start of the study [27]. This treatment “crossing over”, for which we could not account in our models, as well as the potential spill-over effects that occurred with the implementation of DMPs are likely to have biased the mortality effects in the direction of the null. Despite these limitations, non-randomized evaluations like this can be considered as the best available evidence, as randomized designs are hardly possible within the given legal framework [20]. Another limitation of the study is its potentially limited power. Although we observed considerable effect sizes for many of the analyzed associations, tests statistics were often not significant.

## Conclusion

The German DMPs for DM and CHD are among the biggest programs worldwide, but evidence of effectiveness is weak. This study indicates mixed results, speaking weakly in favor of DMPs. However, treatment according to guideline care seems to be highly effective in this patient group. The development of the DMP-guideline care association over time indicates external effects, which should be subject of further investigations. More sophisticated and larger studies are needed to draw more reliable conclusions about the effectiveness of these programs for patients with diabetes and CHD.

## Additional files

Additional file 1: Appendix 1. Adjusted hazard ratios (HR) and adjusted mean differences on the association between dimensions of guideline care and mortality/QoL-change. Additional file 2: Appendix 2. Adjusted odds ratios (OR) on the association between DMP status/guideline care and different dimensions of self-management.
