# Supplementary material for: The effectiveness of German disease management programs (DMPs) in patients with type 2 diabetes mellitus and coronary heart disease: results from an observational longitudinal study
Source: Diabetol Metab Syndr. 2015 Sep 17;7:77. doi: 10.1186/s13098-015-0065-9 (PMC4574141; doi:10.1186/s13098-015-0065-9)
Supplement: Additional file 1: Appendix 1. — Adjusted hazard ratios (HR) and adjusted mean differences on the association between dimensions of guideline care and mortality/QoL-change. [file 13098_2015_65_MOESM1_ESM.docx]

| **Appendix 1:** Adjusted hazard ratios (HR) and adjusted mean differences on the association between dimensions of guideline care and mortality/QoL-change | | | | | |
| --- | --- | --- | --- | --- | --- |
|  |  | Cox regression model | | linear regression model | |
|  |  | mortality | | change in EQ-5D per year | |
| **dimensions of guideline care** | **prevalence (%)** | **HR (yes vs. no)** | **95% CI** | **beta** | **95% CI** |
| advice on diet | 85.9 | 0.57 | (0.30, 1.10) | -0.017 | (-0.041, 0.006) |
| advice on exercise | 81.4 | 0.65 | (0.35, 1.20) | -0.010 | (-0.031, 0.010) |
| advice on smoking | 100.0 | - | - | - | - |
| intake of statins | 78.8 | 0.40 | (0.23, 0.69) | 0.005 | (-0.018, 0.028) |
| intake of beta-blocker | 85.0 | 0.27 | (0.15, 0.48) | -0.008 | (-0.036, 0.019) |
| intake of PAIs ƚ | 84.0 | 0.43 | (0.24, 0.77) | 0.010 | (-0.015, 0.035) |
| intake of RAIs ǂ | 76.9 | 0.69 | (0.38, 1.26) | 0.009 | (-0.010, 0.028) |
| ƚ platelet aggregation inhibitors; ǂ renin angiotensin inhibitors | | | | | |
| models adjusted for age, sex, education, smoking status, weight-status, treatment status, number of re-infarctions and diabetes duration | | | | | |
