# Supplementary material for: The effectiveness of German disease management programs (DMPs) in patients with type 2 diabetes mellitus and coronary heart disease: results from an observational longitudinal study
Source: Diabetol Metab Syndr. 2015 Sep 17;7:77. doi: 10.1186/s13098-015-0065-9 (PMC4574141; doi:10.1186/s13098-015-0065-9)
Supplement: Additional file 2: Appendix 2. — Adjusted odds ratios (OR) on the association between DMP status/guideline care and different dimensions of self-management. [file 13098_2015_65_MOESM2_ESM.docx]

| **Appendix 2:** Adjusted odds ratios (OR) on the association between DMP status/guideline care and different dimensions of self-management | | | | | | | | | |
| --- | --- | --- | --- | --- | --- | --- | --- | --- | --- |
|  | | logistic regression model | | | | | | | |
|  |  | DMP (CHD or DM) | | guideline care | | DMP-CHD (only) | | DMP-DM (only) | |
| **self-management** | % | yes vs. no | | yes vs. no | | yes vs. no | | yes vs. no | |
|  |  | **OR** | **(95% CI)** | **OR** | **(95% CI)** | **OR** | **(95% CI)** | **OR** | **(95% CI)** |
| regular moderate physical activity | 72.7 | 1.81 | (0.80–4.09) | 1.08 | (0.48–2.40) | 1.51 | (0.71–3.19) | 1.43 | (0.66–3.10) |
| regular self-monitoring of blood glucose | 60.3 | 1.09 | (0.43–2.75) | 0.47 | (0.19–1.14) | 1.46 | (0.66–3.23) | 1.09 | (0.47–2.51) |
| regular self-monitoring of blood pressure | 48.0 | 1.23 | (0.60–2.52) | 0.85 | (0.44–1.64) | 1.07 | (0.57–1.99) | 0.93 | (0.49–1.77) |
| regular self-monitoring of body weight | 50.7 | 1.17 | (0.60–2.31) | 0.83 | (0.44–1.57) | 1.31 | (0.72–2.40) | 1.21 | (0.65–2.24) |
| medication adherence | 82.6 | 1.72 | (0.71–4.15) | 0.80 | (0.33–1.95) | 1.40 | (0.62–3.14) | 1.42 | (0.60–3.36) |
| knowing the term "HbA1c" | 72.0 | 3.05 | (1.13–8.20) | 1.49 | (0.59–3.76) | 3.54 | (1.43–8.79) | 2.14 | (0.86–5.30 |
| participation in blood pressure education | 15.2 | 4.68 | (0.88–25.01) | 8.24 | (1.9–35.65) | 4.73 | (1.23–18.14) | 1.82 | (0.58–5.71) |
| participation in diabetes education | 47.4 | 1.59 | (0.69–3.66) | 1.31 | (0.62–2.75) | 2.10 | (1.01–4.40) | 1.69 | (0.80–3.58) |
| DMP: disease management program; CHD: coronary heart disease; DM: diabetes mellitus | | | | | | | | | |
| * counseling on two out of the three lifestyle topics (smoking, diet, exercise) and intake of a platelet aggregation inhibitor and a statin and either renin–angiotensin inhibitors or a beta blocker | | | | | | | | | |
| models adjusted for age, sex, education, smoking status, weight status, treatment status, number of reinfarctions, and diabetes duration | | | | | | | | | |
